# Safety and efficacy of graded dosing of Pfizer-BioNTech mRNA COVID-19 vaccine after an immediate hypersensitivity reaction to first dose

**DOI:** 10.1016/j.jacig.2022.04.005

**Published:** 2022-04-30

**Authors:** Prudhvi Regula, David Rosenstreich, Elina Jerschow, Manish Ramesh, Denisa Ferastraoaru, Jessica Oh, Daniella S. Aivazi, Jonathan M. Aivazi, Golda Hudes

**Affiliations:** a Division of Allergy and Immunology, Department of Medicine, Albert Einstein College of Medicine/Montefiore Medical Center, Bronx, NY; b CUNY School of Medicine, the Sophie Davis School of Biomedical Education Program, New York, NY; c the New York Institute of Technology College of Osteopathic Medicine, Glen Head, NY

**Keywords:** Graded dosing of vaccine, vaccine desensitization, mRNA vaccine allergy, vaccine hesitancy, COVID-19 vaccines

## Abstract

Current guidelines do not recommend subsequent mRNA COVID-19 vaccination in patients who experience immediate allergic reactions to the first dose. Our findings indicate that graded dosing of this vaccine is safe, efficacious, and useful for treating these individuals with allergy.

Emerging data on the recently approved Moderna and Pfizer severe acute respiratory syndrome coronavirus 2 (SARS-CoV-2) mRNA coronavirus disease 2019 (COVID-19) vaccines have demonstrated that they are a critical tool in combating the spread of and mortality and morbidity due to COVID-19 infection.^1^ A large population-based study on non–COVID-19 vaccine reactions reported the incidence of anaphylaxis as 1.3 cases per 1 million administered doses,^2^ whereas the initial reports of mRNA COVID-19 vaccination estimated the incidence of anaphylaxis to be 2.5 to 4.7 cases per million doses.^3^ This higher anaphylaxis rate than that associated with other vaccines^3^ has led to a significant amount of vaccine hesitancy.^4^ Current US Centers for Disease Control and Prevention guidelines state that individuals who have experienced a severe reaction to the first dose are contraindicated from receiving further doses.^5^ Documented preexisting hypersensitivity to excipients, such as polyethylene glycol (PEG) or polysorbate 80, are also contraindications,^5^ although recent studies have shown that the role of excipient skin testing in determining PEG allergy is limited.^6^ There are no clear guidelines on how to proceed with the management of these patients.

A US Centers for Disease Control and Prevention working group has speculated that patients with an adverse allergic reaction to the first dose of mRNA COVID-19 vaccines could consider a subsequent dose of the Janssen adenovirus-based vaccine, but the safety and efficacy of a mixed series have not been adequately studied,^7^ and the thrombosis risk associated with the Janssen vaccine is an additional concern. For patients who develop anaphylaxis after receiving a non–COVID-19 vaccine and need subsequent vaccine doses, the current guidelines recommend graded vaccine administration for those with a positive vaccine skin test result.^8^ However, because mRNA is unstable, there is some concern about the effectiveness of graded administration of this type of vaccine. Currently, the data on the immunogenicity of graded administration of mRNA COVID-19 vaccines in patients who experienced an immediate hypersensitivity reaction to a first dose of mRNA COVID19 vaccine are limited to 1 case series of 15 patients reported by Tuong et al.^9^ This group demonstrated the efficacy of graded dosing by comparing post-graded dosing spike antibody levels with antibody levels in healthy subjects who received both doses of an mRNA COVID-19 vaccine.

We report our experience with graded dosing to a second dose of Pfizer vaccine in patients who experienced an immediate hypersensitivity reaction (within 4 hours) to a first dose of any mRNA COVID-19 vaccine. This was a retrospective observational study approved by the Institutional Review Board at Montefiore Medical Center/Albert Einstein College of Medicine (IRB #: 2021-12803). Ten patients seen at our allergy clinic who met these criteria were identified by retrospective chart review. We compared COVID-19 spike antibody levels before and after administration of the vaccine using graded vaccine dosing. Details of the characteristics of all the patients are described in Table I. Patients were predominantly female (90%) and of diverse racial backgrounds; their mean age was 42 years. Most of the patients (80%) had at least 1 allergic comorbidity. Two patients (20%) had a history of an allergic reaction to influenza vaccine. The Pfizer-BioNTech mRNA COVID-19 vaccine was the first dose vaccine in the majority (70%) of patients. Six patients (60%) had developed anaphylaxis (based on the 2020 World Allergy Organization anaphylaxis criteria), and 4 required epinephrine treatment. Because the Moderna mRNA COVID-19 vaccine was not available at our center, all of these patients, regardless of their first dose vaccine, received the Pfizer mRNA COVID-19 vaccine in incremental doses at 30-minute intervals to a cumulative dose of 0.3 mL (Table II). The protocol used for graded dosing varied owing to physician judgment. All patients were premedicated with a second-generation H1 antihistamine on the day of vaccination, and all patients were observed for 1 hour after the final vaccine dose. COVID-19 spike antibody levels were measured at least 2 weeks after the procedure.

**Table I:** Demographic characteristics of all the patients, details of their first COVID-19 vaccine dose reaction, and its management

PT No.	Age/sex	Race and ethnicity	Allergic comorbidities	History of anaphylaxis	Vaccine allergy	COVID-19 infection	mRNA vaccine used for first dose	Reaction to the first dose of the vaccine	Management of reaction to the first dose	Vaccine excipien*t* test	Pre-desensitization SSIG∗ level (AU/mL)	Post-desensitization SSIG level (AU/mL)	Serum tryptase level (μg/L)
1	29 y/female	White	None	No	No	No	Moderna	Within 5 min: lip and face swelling, dizziness, and presyncopal episode	In ED: epinephrine, steroids, antihistamine	Not done	N/A	N/A	13.7†
2	47 y/female	Hispanic	AR, food allergy	No	Influenza	No	Pfizer-BioNTech	Within 10 min: lip and tongue swelling, shortness of breath	In vaccine center: diphenhydramine	Negative (PEG 3350, methylprednisolone acetate injectable suspension)	N/A	34, 619	N/A
3	24 y/female	White	Asthma, AR	No	No	No	Moderna	Within 25 min: hives, throat closing sensation, severe tongue swelling	In ICU: epinephrine, diphenhydramine, and methylprednisolone	Not done	7657.3	21,513	4.3
4	68 y/female	White	Asthma, AR, shellfish and peanut allergy	Shrimp, peanut	No	No	Pfizer-BioNTech	Within 15 min: throat closing sensation and shortness of breath; similar reaction the next day	In ED: epinephrine, methylprednisolone and diphenhydramine	Negative (PEG 3350)	<50 negative	16,455	6.4
5	33 y/female	African American	AR, food allergy	No	No	No	Moderna	Within 30 min: shortness of breath, tongue and face swelling	In ED: epinephrine, antihistamines and steroids	Not done	73.5	22,954	2.8
6	38 y/female	Hispanic	AR	No	No	No	Pfizer-BioNTech	Within 15 min: rash and itching over the entire body	In vaccine center: diphenhydramine	Negative (methylprednisolone acetate injectable suspension)	N/A	N/A	3.8
7	63 y/female	African American	AR; shellfish, PCN, and contrast media allergy	Contrast media	No	No	Pfizer-BioNTech	Within 5 min: throat itching, nausea, abdominal discomfort, and then dizziness	In ED: diphenhydramine, famotidine, prednisone	Not done	<50 negative	231.5	6.2
8	32 y/male	African American	None	Influenza vaccine	Influenza	No	Pfizer-BioNTech	Within 5 min: difficulty breathing and throat closing sensation	In home-diphenhydramine	Not done	204.5	22,199	N/A
9	35 y/female	Hispanic	Asthma, AR, PCN and ASA allergy	No	No	No	Moderna	Within 5 min: hives and itching over the entire arm	In vaccine center-diphenhydramine	Not done	N/A	>50,000	N/A
10	51 y/female	African American	AR, food allergy	No	No	No	Pfizer-BioNTech	Within 5 min: abdominal pain, nausea, vomiting	In ED: metoclopramide	Not done	524.3	>50,000	6.1

*AR*, Allergic rhinitis; *ASA*, aspirin; *ED*, emergency department; *ICU*, intensive care unit; *N/A*, Not available; *PCN*, penicillin; *PT*, patient; *SSIG*, SARS-COV-2 spike IgG.

∗ Level of SSIG antibodies to mRNA SARS-CoV-2 vaccine was measured by using the AdviseDx SARS-CoV-2 IgG II assay (a chemiluminescent microparticle immunoassay).

† Patient had persistent elevation of serum tryptase level (14 μg/L), with no other associated symptoms and a negative *c-kit* mutation.

**Table II:** Graded dosing protocols for all patients and their outcomes

Patient No.	Sequential doses (mL) of vaccine to complete a cumulative dose of 0.3 mL	Cumulative time (h)	Reactions during graded dosing
1	0.03, 0.04, 0.05, 0.06, 0.06, 0.06	3.5	None
2	0.05-0.25	1.5	None
3	0.03, 0.06, 0.06, 0.06, 0.09	3	None
4	0.05, 0.1, 0.05, 0.1	2.5	Throat itching after second fractional dose of 0.1 mL. Resolved with diphenhydramine, after which protocol was continued
5	0.05-0.10-0.15	2.5	None
6	0.03, 0.06, 0.06, 0.06, 0.09	3	None
7	0.03, 0.06, 0.06, 0.06, 0.09	3	None
8	0.06, 0.24	1.5	None
9	0.06, 0.24	1.5	None
10	0.03, 0.06, 0.06, 0.06, 0.09	3	None

Only 1 patient (10%) developed mild symptoms during graded dosing (Table II), whereas the rest tolerated the procedure without any allergic or adverse reactions. The incidence and severity of allergic reactions during graded dosing (10%) in this patient cohort were lower and milder than those reported by Tuong et al (33%).^9^ Premedication with antihistamine in our cohort may have contributed to this lower incidence of allergic reactions during graded dosing. Post-graded dosing COVID-19 spike antibody measurements were available for 8 patients, and all of those patients exhibited significant antibody levels. These findings confirm the findings of the previously published report of Tuong et al^9^ on the efficacy of graded dosing of mRNA vaccines. Both pre-graded and post-graded dose administration COVID-19 spike antibody levels were available for 6 patients, and all of them demonstrated a significant rise after graded dosing (Table I). Among these patients, 2 patients with undetectable COVID-19 spike antibodies before graded dosing seroconverted, further confirming the efficacy of graded dosing.

In our case series, only 2 patients had excipient skin testing with medications containing PEG; the rest underwent graded dosing without excipient testing. All of the patients in our series tolerated the procedure regardless of testing, suggesting that graded vaccine administration without excipient testing is safe.

Because 2 (or more) doses of these mRNA vaccines are crucial for protection against the SARS-CoV-2 virus, graded dosing is imperative for those experiencing immediate hypersensitivity reactions. Customizing the graded dose protocol on the basis of severity of the prior reaction did not change the outcome. The same protocol can be also used for administrating booster doses to these patients. This graded vaccine dosing regimen should be an effective tool for promoting complete vaccination and booster doses and for mitigation of vaccine hesitancy in patients who had developed an immediate allergic reaction to the first dose.

To our knowledge, this is the first case series to report the safety of graded dosing of COVID-19 mRNA vaccine by using a customized protocol for each patient and also for demonstrating the efficacy of graded dosing by comparing pre-graded and post-graded dose administration antibody levels in patients who had an immediate reaction to the first dose. Our findings confirm and complement the findings of Toung et al.^9^ Health care providers should consider referring patients who experienced a severe, immediate allergic reaction to an mRNA COVID-19 vaccine to allergists for evaluation and possible graded vaccine dosing. Including recommendations for graded COVID-19 vaccine dosing in the guidelines for management of patients with COVID-19 vaccine allergy would be useful for vaccine-hesitant individuals.
